# Non-Alcoholic Fatty Liver Disease: Current Perspectives and Future Direction in Disease pathogenesis, Treatment and Diagnosis

**DOI:** 10.4172/2161-0444.1000e104

**Published:** 2012-08-03

**Authors:** Shaminie Athinarayanan, Wanqing Liu

**Affiliations:** Department of Medicinal Chemistry and Molecular Pharmacology, College of Pharmacy, Purdue University. West Lafayette, IN 47907, USA

**Keywords:** NAFLD, NASH, GWAS, Omics, liver disease

## Abstract

Non-alcoholic fatty liver disease (NAFLD) is one of the most common liver diseases in the world. An important implication of this disease is the progression of the disease to a more complicated condition called non-alcoholic steatohepatitis (NASH) and the wide variety of clinical presentations. Over the past 5 years, remarkable progresses have been made in understanding the genetic basis for the disease. Recent clinical trials in pharmacotherapy for the disease have been encouraging as well. It is anticipated that the integration of the wide spectrum information retrieved from genomics, transcriptomics and proteomics studies conducted in NAFLD and NASH will mediate a better understanding of the disease pathogenesis and facilitate the postulation of disease pathobiology pathways. Genetic and biological markers identified from the omics studies may hold promise for diagnosis, personalized treatment, early prevention and new drug development.

## Overview

Liver disease is a broad term for any kind of disease manifestation or condition that affects the liver and its function. Being the largest solid organ in the human body, the liver is considered a vital organ with multiple functions, which include detoxification, protein synthesis, metabolism, glycogen storage and decomposition of blood cells. Any disease of this organ may manifest itself in a broad range of symptoms that affect different mechanisms and pathways. This has been well demonstrated in an emerging metabolic liver condition known as non-alcoholic fatty liver disease (NAFLD). The manifestation of NAFLD usually encompasses a broad spectrum of symptoms and clinical presentation ranging from simple steatosis to nonalcoholic steatohepatitis (NASH), which eventually may progress to liver cirrhosis and/or hepatocellular carcinoma (HCC) [1–3]. NAFLD is one of the most common liver diseases in the world with a prevalence of 20–30% in the United States [1,3] and approximately 15% in the Asian population [3]. This disease has also been reported among children with an estimate of 3 to 10%, and a higher prevalence has been observed among obese children [1]. It is well-established that NAFLD is linked to obesity, insulin resistance and metabolic syndrome [1,3]. However, due to the presentation of the disease condition and the wide range of clinical presentation of the disease, there is no clear delineation of the disease pathogenesis. It has been elucidated that this disease has a multifactorial emergence and a genetic predisposition due to the interethnic variations observed [4,5] and higher preponderance within families [6,7]. The two-hit hypothesis which was initially proposed in 1998 [8] has been widely used as the pathogenesis pathway of steatosis (first hit) and progression of steatosis (second hit) to NASH. This hypothesis seems to correlate with some of the biochemical and genetic studies conducted on NAFLD [9]. Liver biopsy and histology have long been identified as the “gold standard” method for diagnosing and confirming NASH. This is the only test used to accurately assign the different disease stages that can be used as a prognostic indicator of the disease condition. However, the invasiveness of the procedure and the susceptibility of sampling error limit the usefulness of this method for routine diagnostic. Most clinicians and physicians utilize the diagnosis of exclusion approach instead, for ruling in NASH and NAFLD. Even though this approach is not directly useful in confirming NASH, it has been extensively used as predictors for further evaluation and biopsy request. Alternatively, different radiographic imaging techniques have served to identify the presence and extent of steatosis in the liver of NAFLD patients.

Many different treatment options have been tested for NAFLD and NASH thus far. However, it was not until very recently that consistent pharmacotherapy strategy for the disease was possible. One of the most frequently recommended treatment regimens for patients diagnosed with NAFLD is to lose excess weight, especially in cases with obese or overweight patients. Several early small trials have demonstrated the effectiveness of vitamin E and C in treating NAFLD in adults and children [10,11]. These studies initiated the two large, standardized random clinical trials in adults and children [Pioglitazone versus Vitamin E versus Placebo for the Treatment of No diabetic Patients with Nonalcoholic Steatohepatitis (PIVENS) and Treatment of Nonalcoholic Fatty Liver Disease in Children (TONIC)] to demonstrate the usefulness of vitamin E as a therapeutic agent for treating NASH and NAFLD [12,13]. Both studies showed a promising effect on improving NASH clinical and histologic symptoms in adults and children, though failed to demonstrate significant outcome in improving the alanine aminotransferase (ALT) level in children. This finding sheds new light on the pharmacotherapy and early prevention of NAFLD and NASH.

## Current Perspectives

Increasing awareness of the disease manifestation, emergence of the disease among pediatric patients, and the importance of detecting this disease in its early stage have molded more genetics and omics related research for understanding the pathogenesis of NAFLD and NASH. Although numerous studies focusing on candidate genes and pathways have been conducted in the past decades, most findings remained contradicting. Recent genome-wide association studies (GWAS) in large patient populations have resulted in a great leap forward to understanding the natural history of NAFLD and NASH. The study by Romeo et al. [14] demonstrated a very strong association between hepatic fat content, hepatic inflammation, and a variant (rs738409) of PNPLA3 gene, which results in the substitution of isoleucine to leucine in this gene product. Although the specific effect of this gene in the liver seems vague and more studies are currently in place to understand the role of this gene in lipid metabolism, this susceptibility allele has been broadly confirmed independently and has been associated with a wide spectrum of the phenotypes in NAFLD and NASH [15,16]. Additional GWAS studies focusing on NAFLD phenotypes defined with different approaches have also identified more risk alleles in NCAN, PPP1R38, GCKR, LYPLAL1, FDFT1, COL13A1, LTBP3, EFCAB4B, ZP4, PZP and DDX60L/PALLD genes [16,17]. Besides these GWAS, other omics analyses using high-throughput transcriptomic, proteomic, metabolomic and lipidomic technologies also made a great progress in identifying pathways involved in the pathogenesis of the disease as well as potential diagnostic markers. Transcriptomic studies were able to demonstrate the differential expression of mRNAs correlating with the different NAFLD clinical spectrum even though the hepatic expression pattern slightly differed between the studies [18,19]. Different analysis platforms and/or sample types were utilized for all the different proteomic studies on NAFLD to date and yet these studies were able to demonstrate marked variations in the protein profiles between different spectrums of NAFLD [20–23]. Some of these studies were successful in distinguishing distinct protein markers that were suggested as possible diagnostic markers. Charlton et al. [22] reported the significance of keratin sulfate proteoglycan over expression directly correlating with progressive NASH symptoms and another protein marker, fatty acid binding protein-1 (FABP-1) showed reduced expression in NASH when compared to its expression in simple steatosis. Another study by Gray et al. [23] identified five significant protein markers that correlated with NAFLD associated fibrosis and HCC and suggested the usefulness of these proteins as possible diagnostic markers. Lipidomics studies in NAFLD were successful in demonstrating the involvement of peroxisomal polyunsaturated fatty acid (PUFA) metabolism [24] and free radical mediated linoleic acid oxidation [24,25] with NASH and the disease progression. Plasma metabolomics study showed strong association between bile salts and glutathione metabolites with NAFLD samples [26]. Data collected from all these different omics studies together strongly suggest the involvement of multiple metabolic and pathogenesis pathways in NAFLD.

## Future Directions

It is anticipated that the integration of the wide spectrum of information retrieved from genomics, transcriptomics, metabolomics, lipidomics and proteomics studies conducted in NAFLD and NASH will mediate future unequivocal interpretation of these multitudes of data for understanding the disease pathogenesis and clinical variation. This definitely emphasizes the importance of open access availability of research information and ability to interact between different research areas. One of the key elements for future research direction may be the identification of the linkage between significant genes, transcripts, lipids, metabolites and proteins identified from omics research and to finally determine the key pathobiology network and pathway involved in the development of hepatic steatosis, initiation of hepatic inflammation and fibrosis, transition to NASH, and progression to liver cirrhosis and hepatocellular carcinoma. Significant genetic and biological markers, especially those involved in a specific signaling pathway, can be used for novel drug target development in the future. Moreover, the integrated information may also provide a better understanding of the drug response variation in NAFLD and NASH and form the solid basis for pharmacogenomics-based studies on specific drugs used for treating NAFLD and NASH. The question remains whether the polymorphisms in some of the significant drug targets and metabolism genes directly associated with NAFLD pathogenesis and drug metabolic genes can be correlated with a specific drug response, especially drugs that have shown promising therapeutic intervention such as Vitamin E. In addition, future research in the development and validation of non-invasive diagnostic tissue markers or biomarkers, for example, carrying of PNPLA3 risk allele for early intervention, might represent another area of scope to focus. These important markers can be used for diagnosing NAFLD and NASH less or non-invasively and/or be useful predictors for drug administration and drug response monitoring. This is only possible if the biochemical role of PNPLA3, the specific PNPLA3 substrate and the role of this gene in NAFLD development were accurately investigated. Furthermore, other prospective omics research areas which will benefit the understanding of NAFLD pathogenesis include metabolomic and lipidomic analysis for both human samples and samples from animal models. This field of study, combined with the genetic information may ultimately lead to personalized nutrition strategies for early prevention and dietary treatment for both NAFLD and NASH.
